# An Algorithm for Tracking the Position and Velocity of Multiple Neuronal Signals Using Implantable Microelectrodes In Vivo

**DOI:** 10.3390/mi12111346

**Published:** 2021-10-31

**Authors:** Lionel M. Broche, Karla D. Bustamante, Michael Pycraft Hughes

**Affiliations:** 1 Centre for Biomedical Engineering, University of Surrey, Guildford GU2 7XH, UK; l.broche@abdn.ac.uk (L.M.B.); karla.bustamante@cibac.mx (K.D.B.); 2 Aberdeen Biomedical Imaging Centre, University of Aberdeen, Foresterhill, Aberdeen AB25 2ZD, UK; 3 Centro de Investigación en Bioingeniería A.C., 4709 Avenida H. Colegio Militar, Chihuahua 31150, Mexico

**Keywords:** neural probe, neuroprobe, recording, implant

## Abstract

Increasingly complex multi-electrode arrays for the study of neurons both in vitro and in vivo have been developed with the aim of tracking the conduction of neural action potentials across a complex interconnected network. This is usually performed through the use of electrodes to record from single or small groups of microelectrodes, and using only one electrode to monitor an action potential at any given time. More complex high-density electrode structures (with thousands of electrodes or more) capable of tracking action potential propagation have been developed but are not widely available. We have developed an algorithm taking data from clusters of electrodes positioned such that action potentials are detected by multiple sites, and using this to detect the location and velocity of action potentials from multiple neurons. The system has been tested by analyzing recordings from probes implanted into the locust nervous system, where recorded positions and velocities correlate well with the known physical form of the nerve.

## 1. Introduction

Since the first development of action potential measurement in the 1950s [1], instruments have been developed to enable the monitoring of single action potentials (APs). Whilst original recording devices comprised thin wires, since the 1970s, the predominant approach to neural recording electrodes has been through the use of micromachined silicon electrodes constructed using techniques developed in the semiconductor industry. As manufacturing techniques have improved, it has been possible to increase the number of electrodes on each device in order to enable recording from multiple neurons simultaneously, either in in vivo or in vitro settings [2,3,4,5]. When recording from complex structures containing many neurons, it is important to be able to discriminate between different neural sources. Where single electrodes are used in multi-neuron settings, this is commonly performed by classifying the sources according to amplitude to determine proximity, though this is only effective for AP sources adjacent to the electrode; as distance increases, so does the likelihood of multiple sources being at similar distance from the electrode in any given direction; such single electrode systems (or multi-electrode systems with inter-electrode spacings of hundreds of microns) are unable to give spatial information about these sources, such as location or velocity. Pickard et al. demonstrated that a single implanted multi-electrode probe can detect many action potentials by comparing the relative magnitudes of the potential “spike” [6]. This approach has been used by other groups to discriminate between APs, but the typical wide spacing between microelectrodes of implantable devices (typically multiple hundreds of microns) means that AP spikes can be detected by only one electrode at a given point in time (e.g., [7]).

Where spatial information such as AP location, detection and velocity are required, multiple electrodes must be used. A common approach to this is to track APs using large multiple-electrode arrays (MEAs), such that individual APs pass near many electrodes and can consequently be identified, tracked and measured. However, such MEAs typically use high levels of redundancy, with many electrodes (sometimes extending to hundreds) required to track each AP, and each electrode recording from a small number of sources [8]. Whilst in vitro MEAs can be manufactured to fit the size of a brain slice or cultured neural structure, in vivo devices are more limited in order to maximize proximity to neuron sources without causing significant damage to the neural tissue. Consequently, for in vivo electrodes, a more efficient approach to detection of APs is required, using smaller numbers of electrodes in close proximity to one another and use triangulation techniques in order to identify multiple AP sources. This has been used to measure the position of multiple sources in two dimensions using parallel metal wires [9], though the nature of the very long electrodes placed parallel to the direction of neural processes means the approach is only able to classify forces radially, and relies on the electrode direction being completely parallel to the neural sources. Another approach developed by Hughes et al. [10] used point electrodes to measure action potential velocity by measuring the time taken to travel between electrodes placed along the nerve.

In this paper, we present an approach to detecting action potentials from multi-unit recordings that allow us to track the axon source across a nerve with as few as three electrodes by triangulating the signals from multiple electrodes, and comparing their timings and amplitudes. In order to evaluate the usefulness of the algorithm, we have validated the model by applying it to signals recorded using a microelectrode device implanted into a locust ventral nerve cord, a complex network of non-myelinated nervous systems with defined physical dimensions and with action potential travelling along a defined afferent-efferent direction of travel. The result demonstrated a clear correlation between the analyzed results and the nerve geometry. This has the potential to provide a new approach to multi-unit neural recordings, simplifying the production of electrode arrays for the analysis of very large numbers of neurons without the requirement for commensurately large numbers of electrodes.

## 2. Detection Algorithm

We aimed to develop a method of AP tracking that minimizes the number of electrodes required for neural monitoring, by using three electrodes arranged on a penetrating probe with a maximum distance between the furthest electrodes of less than 100 µm. These were used not only to identify individual APs, but also to calculate their position, direction and velocity. The analysis approach is derived from a model of the movement of an AP past multiple electrodes.

**Figure 1:**
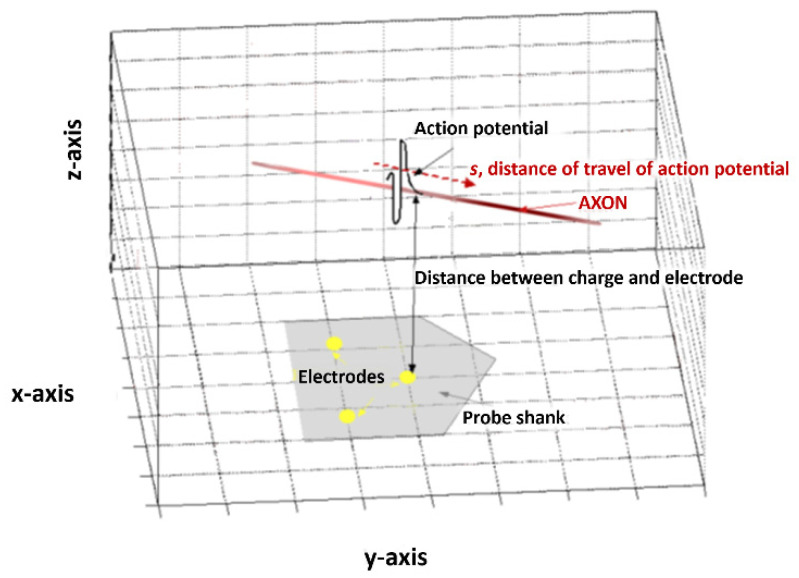
A schematic representation of the model used in this processing technique. An action potential with vector S travels with respect to multiple electrode sites (here shown on a schematic of a neural probe, in grey).

In order to explain the analysis method, we will describe the model on which it is based, which can be seen in Figure 1. At its simplest, we can model an AP as a charge *Q* travelling in a linear axon parallel to the probe surface. From Coulomb’s law, the potential caused by a charge at a point *a* will be inversely proportional to the distance between that point and the charge. At the scale of the probe, a nerve can reasonably be considered linear and the position of the probe can be made as to satisfy the condition of parallelism.

As our electrodes are in planar arrays, we can define them purely in the *x*–*y* plane. We can simplify the electrodes by considering them as a recording point with position ${\vec{M}}_{i}=x_{i}.{\vec{u}}_{x}+y_{i}.{\vec{u}}_{y}$. The potential at each electrode at time *t* depends on the distance *D_i_* between the charge and the electrode, which can be expressed as a function of the position *s* of the charge along the axon and on the minimum distance between the electrode and the axon. Taking into account the AP velocity $\vec{v}=v_{x}.{\vec{u}}_{x}+v_{y}.{\vec{u}}_{y}$, one obtains the following:

(1) $${\vec{D}}_{i}=-{\vec{M}}_{i}+{\vec{s}}_{o}+t\cdot \vec{v}$$

where

${\vec{s}}_{0}=x_{0}.{\vec{u}}_{x}+y_{0}.{\vec{u}}_{y}+z_{0}.{\vec{u}}_{z}$ is a reference point on the axon defined by the position of the AP at time 0.

The potential at the electrode reaches its maximum value when the charge is at the point of the axon closest to the probe. We call this point ${\vec{s}}_{m,i}$ with coordinates $x_{m,i},\;y_{m,i},\;z_{m,i}$, where *m* corresponds to ‘minimum’ and *i* describes reference number of the electrode. Since the probe is parallel to the axon, one can write:

(2) $${\vec{M}}_{i}\cdot {\vec{s}}_{m,i}=0=\left(x_{m,i}-x_{i}\right)\cdot t\cdot v_{x}+\left(y_{m,i}-y_{i}\right).t.v_{y}$$

We can now introduce the time $t_{i}$ taken for the action potential to reach point ${\vec{s}}_{m,i}$ from the reference ${\vec{s}}_{0}$ related to the position of the action potential at time 0 so that:

(3) $$\begin{array}{c} x_{m,i}=x_{0}+t_{i}\cdot \upsilon_{x}\\ y_{m,i}=y_{0}+t_{i}\cdot \upsilon_{y}\\ z_{m,i}=z_{0} \end{array}$$

By combining the equations above, it is possible to find the position of the minimum approach point ${\vec{s}}_{m,i}$ in terms of the velocity, the electrode coordinates (*x_i_*,*y_i_*) and the arbitrary reference point (*x_o_*,*y_o_*); the z-coordinate is invariant due to its orthogonal orientation to the plane of the electrodes.

(4) $$\begin{array}{l} x_{m,i}=\frac{x_{i}\cdot v_{x}{}^{2}+v_{x}\cdot v_{y}(y_{i}-y_{o})+x_{o}\cdot v_{y^{}}{}^{2}}{v_{x}{}^{2}+v_{y}{}^{2}}\\ y_{m,i}=\frac{y_{i}\cdot v_{y}{}^{2}+v_{x}\cdot v_{y}(x_{i}-x_{o})+y_{o}\cdot v_{x}{}^{2}}{v_{x}{}^{2}+v_{y}{}^{2}} \end{array}$$

The coordinates for the electrode *i* and the origin are known, but not the velocity or the position of the reference point. We can use the information provided by another electrode, say electrode *j*; by introducing $\Delta x_{ij}=\left(x_{m,i}-x_{m,j}\right)$, $\Delta y_{ij}=\left(y_{m,i}-y_{m,j}\right)$ and $\Delta t_{ij}=\left(t_{i}-t_{j}\right)$ one obtains, after some calculations:

(5) $$\Delta t_{ij}\cdot (v_{x}{}^{2}+v_{y}{}^{2})=v_{x}\cdot \Delta x_{ij}+v_{y}\cdot \Delta y_{ij}$$

Equation (5) has two unknown variables, *v_x_* and *v_y_*, and therefore one more electrode (here terms electrode *k*) is required. Using Equation (5) for both pairs of electrode gives the following:

(6) $$\begin{array}{l} \frac{v_{x}}{v_{x}{}^{2}+v_{y}{}^{2}}=\frac{\Delta t_{ij}\cdot \Delta y_{ik}-\Delta t_{ik}\cdot \Delta y_{ij}}{\Delta y_{ij}\cdot \Delta x_{ik}-\Delta x_{ij}\cdot \Delta y_{ik}}=K_{x}\\ \frac{v_{y}}{v_{x}{}^{2}+v_{y}{}^{2}}=\frac{\Delta t_{ij}\cdot \Delta x_{ik}-\Delta t_{ik}\cdot \Delta x_{ij}}{\Delta y_{ij}\cdot \Delta x_{ik}-\Delta x_{ij}\cdot \Delta y_{ik}}=K_{y} \end{array}$$

It is useful at this stage to introduce the following complex number:

(7) $$K_{x}-i.K_{y}=\frac{v_{x}-i.v_{y}}{v_{x}^{2}+v_{y}^{2}}=\frac{1}{\left(v_{x}+i.v_{y}\right)}$$

Such that:

(8) $$v_{x}+i.v_{y}=\frac{1}{K_{x}-i.K_{y}}$$

This complex variable can provide the velocity of the action potential by taking the real and imaginary part of its inverse. The values *K_x_* and *K_y_* depend on two variables: the position of the electrodes, and the difference in time between the observation of the maximum signal of an AP at the two different electrodes, assuming that the probe is inserted such that the general direction of neural travel is in the direction of the *x*-axis, and that the difference in times of an action potential seen by two electrodes can be calculated from the recordings. It is therefore possible to process *K_x_* and *K_y_* and to estimate the velocity; one can also determine the position of the neuron by using the model of propagation of the electric field, since the potential due to a charge *q* is given by the expression

(9) $$V_{i}(t)=\frac{1}{4\pi \varepsilon \varepsilon_{0}}\frac{q}{D_{i}}=\frac{q\prime }{D_{i}}$$

where *q′* = *q*/4*πεε*_0_. This expression is difficult to solve in this form, but can be simplified by using a local frame of reference {*X*, *Y*, *Z*} rotated by an angle $\alpha =\arctan(v_{y}/v_{x})$, such that the neuron is aligned with the *X*-axis. This reduces the number of unknowns to three, the coordinates *Y* and *Z* and the charge *q*. This can be further simplified by considering the potential *V_i_* for each electrode when the action potential is at the point ${\vec{s}}_{m,i}$ so that no coordinates along the *X*-axis appear in the equations. Under these conditions, combining Equations (1) and (9) for all the electrodes in the local frame of reference provides the following solution:

(10) $$\left\{ \begin{array}{l} q^{2}=\frac{\left(Y_{i}^{2}\left(Y_{j}^{}-Y_{k}^{}\right)+Y_{j}^{2}\left(Y_{k}^{}-Y_{i}^{}\right)+Y_{k}^{2}\left(Y_{i}^{}-Y_{j}^{}\right)\right)}{S_{i}^{-2}(Y_{k}^{}-Y_{j}^{})+S_{j}^{-2}(Y_{i}^{}-Y_{k}^{})+S_{k}^{-2}(Y_{j}^{}-Y_{i}^{})}\\ Y=\frac{1}{2}\frac{S_{i}^{-2}(Y_{k}^{2}-Y_{j}^{2})+S_{j}^{-2}(Y_{i}^{2}-Y_{k}^{2})+S_{k}^{-2}(Y_{j}^{2}-Y_{i}^{2})}{S_{i}^{-2}(Y_{k}^{}-Y_{j}^{})+S_{j}^{-2}(Y_{i}^{}-Y_{k}^{})+S_{k}^{-2}(Y_{j}^{}-Y_{i}^{})}\\ Z^{2}=\frac{q^{2}}{S_{i}^{2}}-{\left(Y_{}-Y_{i}\right)}^{2}=\frac{q^{2}}{S_{j}^{2}}-{\left(Y_{}-Y_{j}\right)}^{2}=\frac{q^{2}}{S_{k}^{2}}-{\left(Y_{}-Y_{k}\right)}^{2} \end{array}\right.$$

where the variables *S_i_, S_j_* and *S_k_* represent the maximum amplitudes of an aAP seen by electrodes *i*, *j* and *k* at positions *Y_i_*, *Y_j_* and *Y_k_*. The coordinates *Y* and *Z* are expressed in the local frame and must be rotated back to the initial frame.

This preliminary model is made using an assumption that the AP can be represented by a single point charge, which is a coarse estimation. It is possible to improve this by introducing the shape of the action potential. We can consider the action potential as a distribution of charges *Q* along the neuron, or, $Q\prime =Q/4\pi \varepsilon \varepsilon_{0}$ which is a function of time *t* and position *s*. Equation (9) then becomes:

(11) $$\begin{array}{l} V_{i}(t)=\int_{neuron}\frac{Q\prime (t-s/v)}{D_{i}(s)}ds\\ =\int_{neuron}\frac{Q\prime (t-s/v)}{D_{m,i}\sqrt{1+{\left(s-s_{m,i}\right)}^{2}/D_{m,i}{}^{2}}}ds \end{array}$$

where $D_{m,i}$ is the minimum distance between probe *i* and the neuron. Mathematically, Equation (11) can be regarded as a convolution between the charge function *Q′* and a function *F* defined by:

(12) $$F(s)=\frac{1}{\sqrt{1+{\left(\frac{s-s_{m,i}}{D_{m,i}{}^{2}}\right)}^{2}}}$$

Then, Equation (11) becomes:

(13) $$V_{i}\left(t\right)=\frac{1}{D_{m,i}}\left(Q\prime F\right)\left(t\right)=\frac{q_{e,i}\prime \left(t\right)}{D_{m,i}}$$

where

(14) $$q\prime_{e,i}(t)=\int Q\prime (t-s/v).F(s)ds$$

Equation (13) for *V_i_* is similar to Equation (9) for a point charge, but here the equivalent charge depends on the position of the electrode and time. This makes the analytical calculation of the electric potential much more difficult. However, using Equation (12), it is possible to avoid this problem by estimating the first-order error provided by the point-charge approximation. One can note the following:

(15) $$\frac{\partial q_{e,i}\prime (t)}{\partial D_{m,i}}=\int_{s}Q(t-s/v)F(s)\frac{1}{D_{m,i}}\frac{1}{1+D_{m,i}^{2}/{\left(s-s_{i}\right)}^{2}}ds$$

We can set an upper bound to the value of the fraction under the integrand as follows:

(16) $$\frac{1}{1+D_{m,i}^{2}/{\left(s-s_{i}\right)}^{2}}<\frac{1}{1+D_{m,i}^{2}/s_{\max}{}^{2}}$$

And then:

(17) $$\left|\frac{\partial q\prime_{i}(t)}{\partial D_{m,i}}\right|<\frac{1}{D_{m,i}}\frac{1}{1+D_{m,i}^{2}/s_{\max}{}^{2}}\left|q\prime_{i}(t)\right|$$

*s*_max_ can be estimated from the value of the velocity and the AP duration using a plot of function *F*, then one can express the first-order Taylor series of the electric potential as:

(18) $$V_{i}(t)\approx \frac{q\prime (t)}{D_{m,i}}\left(1+\frac{1}{D_{m,i}}\frac{1}{1+D_{m,i}^{2}/s_{\max}^{2}}\left|D_{i}-D\right|\right)=\frac{q\prime (t)}{D_{m,i}}\left(1+\varepsilon_{i}\right)$$

The error *ε_i_* can be processed from the estimated values in order to estimate the error in the position of the neuron.

The resolution of the system is defined by the sampling rate and electrode spacing; for example, if the signals are sampled at 40 kHz, then only differences in time higher than 25 μs can be detected; if in this instance the distance between electrodes is 80 μm then the maximum detectable velocity would be 3.2 m s^−1^, which is sufficient for non-myelinated nervous systems, such as neural cultures or invertebrate study. A 1 MHz, 100 μm system would be required to detect the fastest 100 m s^−1^ action potentials.

## 3. Methods

In order to test the model, we analyzed recordings from implantable neural probes inserted into the ventral nerve cords of desert locusts. The locust model was selected due to the fact that it allowed recordings from a large number of unmyelinated neurons organized in a structure that is both spatially limited (to the dimensions of the cord) and directionally well defined (with axons generally running along the cord axis). The approach was used with a variety of triode penetrating electrode arrays, with electrodes in triangular arrays ranging between 20 and 80 µm apart and with areas between 64 and 177 µm^2^ [11,12,13], manufactured with recording surfaces in either gold or iridium. Past investigation of the effects of varying electrode size and positioning suggests our electrodes were suitable for positioning, being sufficiently small to avoid averaging of the peak in transit, and large enough to minimize errors due to electrode impedance [14].

The probes were mounted into a headstage amplifier with further remote amplification stages being applied before sampling; details of the recording setup can be found elsewhere [15]. The signal was acquired using an AT-MIO 16-F National Instruments acquisition (Austin, TX, USA) card at 40 kHz/channel using a LabView (National Instruments, USA) and saved for later analysis. Recordings were taken from the ventral nerve cord, metathoracic ganglia and jumping leg nerve of the desert locust (L. Migratoria). The experimental protocol for recording can be found elsewhere [15]; experiments were performed in compliance with the relevant laws and University of Surrey guidelines.

Locusts were anaesthetized, decapitated and dissected to expose the cavity where the nerve cord was found, which was filled with locust saline [12] at room temperature. The probe was inserted into the nerve using a micromanipulator such that the electrode plane was parallel to the direction of the nerve. If no spontaneous signals were observed, a gentle touch was applied to the leg, or warm saline (at 28–30 °C) was poured over the body. When the signals started appearing, recordings were taken.

**Figure 2:**
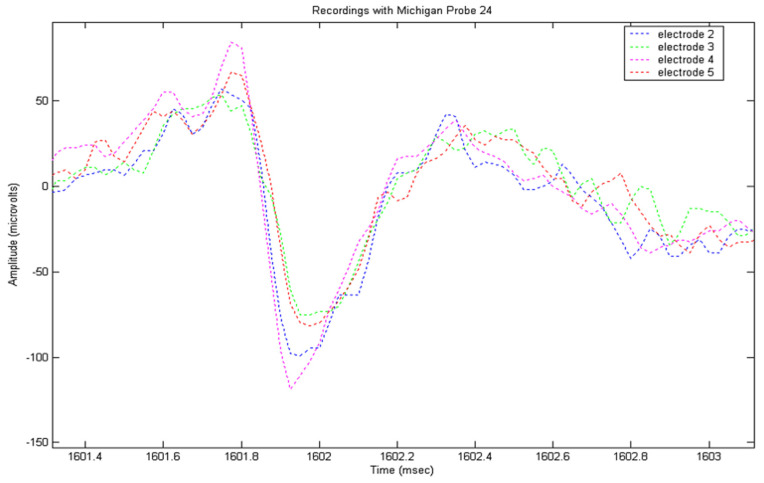
A 4-channel recording using four electrodes arranged in a square, 35 µm along a side, showing different spike amplitudes and peak times according to position.

The recorded signals from the locust model were input into a MatLab (the Mathworks, Natick, MA, USA) program to implement the detection routine described above. Prior to analysis, frequencies below 100 Hz were removed from the signal using a FFT function, followed by wavelet de-noising using the wavelet sym5, selected for its resemblance to an action potential. A total of eight recording sessions were performed; during each of which, significant numbers of APs were recorded. Following analysis, nerves were examined histologically and measured. An example of an AP appearing in multiple electrode channels with different amplitudes and peak times can be seen in Figure 2. Peaks were detected by thresholding, and then tracked through multiple frames, with the amplitude and duration being extracted for each electrode.

## 4. Results and Discussion

**Figure 3:**
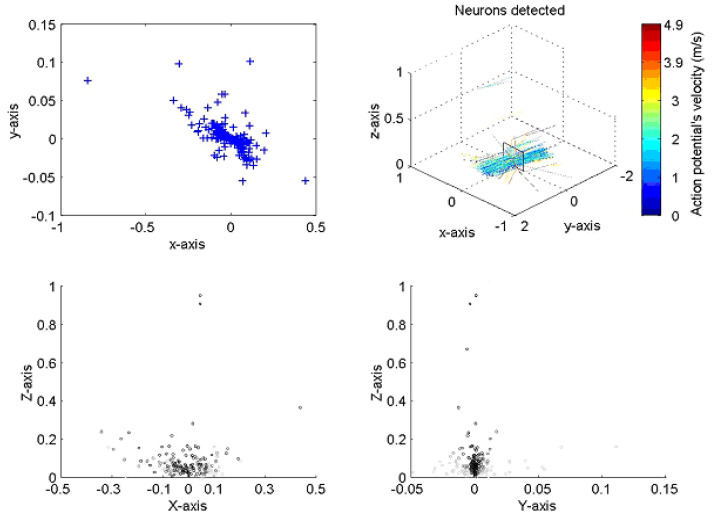

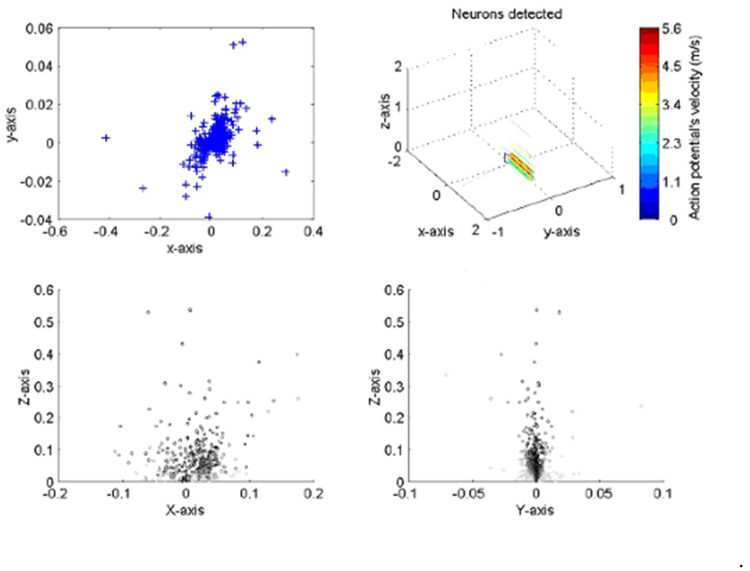
Presentation of two sets of results for the neural processing program when applied to a locus ventral nerve cord. The positions of action potential closest approach in the X–Y axis (top left) corresponds closely to the dimensions of the nerve cord; the velocity and direction correspond well with both previously published work and with the anatomy of the cord. All dimensions in mm.

The implementation of the algorithm is shown in Figure 3 for a typical neural recording. On the top right the figure is a 3D diagram representing the space above the probe. The probe, as illustrated, sits on the *x*–*y* plane pointing towards the positive part of the y axis. The line crossings represent the position of the axons with color indicating the velocity of the action potentials corresponding to a given axon. The plane on top of the probe is a transverse section of the space above the probe where neurons are crossing. The box shows a section of the nerve, the top left and bottom diagrams present the different views of nerve. The top left is a superior view; the bottom left a transverse section and the bottom right a lateral view. The blue crosses represent the position of the neurons at closest approach to the probe. In the analysis, the large majority of lines are running parallel to the probe, indicating that the probe was inserted with an angle parallel to the nerve, as was indeed the case.

We found that with a small percentage (typically 5–10% across multiple experiments) were observed to lie at distances outside the estimated boundary of the ventral nerve cord as estimated using histology. We found that a similar number showed estimated velocity vectors that were at significant variance from the majority. It should be noted that in such cases, greater distance from the probe is determined by smaller signal amplitude, which in turn means the signals are more susceptible to noise.

**Figure 4:**
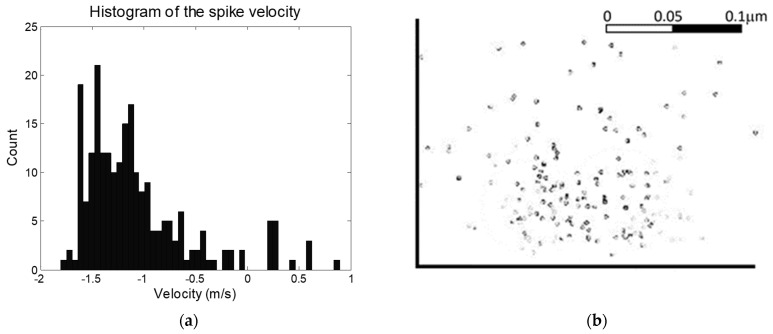
(**a**) A histogram of action potential velocity. The values closely correspond to published data, and the bias in one direction is in keeping with potentials evoked in a decapitated locust, with afferents outnumbering spontaneously-generated efferents. (**b**) Distribution of locations of nearest approach to the electrodes, as a cross-section of the nerve. Darker spots indicate multiple signals.

The locust ventral nerve cord is well characterized in terms of the distributions if neurons of different diameter [15,16,17,18,19,20]. Since action potential velocity varies with neuron diameter, this gives an indication of the distribution of anticipated action potentials, and provides a method of assessing whether the system provides results in line with expectations. The distribution of measured velocities for a typical experiment can be seen in Figure 4. It should be noted that given the nature of the experiment (decapitation followed by stimulation of the leg) that we would anticipate that only afferent signals (heading to the brain) would be generated, with no efferents (in the direction of signals coming from the brain) except reflex arcs. This is in line with observations, in which 93% of signals were observed in one direction. This approach can be expanded to include AP velocity. In an average locust ventral nerve cord [19], 64% of neurons have diameters greater than 4 μm and corresponding velocities greater than 1 ms^−1^; 24% have diameters between 2 and 3 μm and velocities of 0.5–1 ms^−1^; and 12% have diameters below 2 μm and velocities below 0.5 ms^−1^. Our figures showed 74% having greater than 1 ms^−1^, 15% having velocity between 0.5 and 1 ms^−1^; and 11% with velocities below 0.5 ms^−1^. This is in general good agreement with expectations, but suggests the system is more sensitive to large-diameter, rapid neurons than small-diameter, slower neurons. It could be noted that visual inspection of the curve on Figure 4 suggests an unusual spike at ca. 1.2 ms^−1^, which may be attributable to significant numbers of APs from a single neuron skewing the results, and without which the measured and estimated numbers would be more alike. Alternatively, the discrepancy may be attributed to different size ratios being used for afferents vs. efferents, since only the former will be present here (to any significant degree), which are not accounted for in the anatomical studies described above. Examination of the size distribution of a typical analysis (Figure 4b) reveals capture across a radius of approximately 100 µm, but also highlights multiple collocated spikes (indicated by darker markers). This may have an impact on the velocity distribution in Figure 4a, but also suggests the system is effective in classifying neural sources by observing multiple firings at similar special locations.

There are a number of issues that affect the accuracy of the approach. We have investigated variability according to electrode size [14] and fabrication material [21] and found that variability in these over the ranges we have considered does not significantly affect the signals acquired by these electrodes.

The proposed algorithm uses a simple model of field propagation that does not account for discrepancies that might be caused by differences in the tissue environment or the electrical properties of the extracellular medium [22,23]. As outlined above, the necessity for identifying the peak makes the system susceptible to electrical noise, particularly where the AP is relatively small (such as at a distance from the probe, or where the neural diameter is small). Whilst the effect will also be present for signals that pass near the electrode but start at a distance from it, such errors can be eliminated by identification of the signal at closest approach. Conversely, classification by monitoring the AP as it moves through space and time offers advantages over methods that relay on a “snapshot” of nearby APs; for example, whilst the shapes of APs will be different at different positions in space (electrodes). The use of “point” electrodes (ca. >20 µm across) reduces the effect of “flattening” observed with larger electrodes, making the identification of peak timing easier, whilst the taking of multiple frames of the same AP makes identification of the true “peak” detection down to a fraction of a millisecond simpler. This offers significant benefits over “traditional” spike classification studies that only use a single electrode and use amplitude and shape characteristics to sort spikes to specific neurons

However, given the reach of the algorithm (taking a snapshot across a region ca. 200 µm in diameter as seen in Figure 4b), it may be more applicable to the detection of whole nerve patterns rather than identification of individual neurons, enabling the use of pattern recognition to identify patterns relating to specific functions. Such approaches are in common use in the central nervous system for the identification of intention [24,25,26,27]; our technology would enable a similar approach to be taken in both central and peripheral nervous systems. It can also be noted that although the system presented here is designed for detection of APs in non-myelinated nervous systems, the principles of triangulation using multiple electrodes simultaneously tracking APs by amplitude and length has application in the identification of nodes of Ranvier in the region of detection for application in myelinated systems. Alternatively, the work demonstrates that multiple electrodes can be used to track multiple AP sources in, for example, a planar electrode array for in vitro neural analysis, particularly where an interlayer (such as a thin gel) could be used to increase the triangulation effect by increasing the z-dimension between electrode and neuron planes.

## 5. Conclusions

We have developed a model that can be used to track the movement of action potentials in a non-myelinated nervous system. The model developed has been applied to the detection of action potentials in a locust nerve, in which it has detected the positions of the neurons with some success when compared with known anatomy of the nerve. Once fully developed, such a system may also be applied to the more complex issue of tracking action potentials in myelinated nerve tissue, with evident applications in neural recording for interfacing with prostheses.

## Data Availability

Data are available from the authors on request.

## References

[1] Hodgkin A.L., Huxley A.F., Katz B. (1952). Measurement of current-voltage relations in the membrane of the giant axon of *Loligo*. J. Physiol..

[2] Bendali A., Bourguelia S., Roupioz Y., Forster V., Mailley P., Benosman R., Livache T., Sahel J.-A., Picaud S. (2014). Cell specific electrodes for neuronal network reconstruction and monitoring. Analyst.

[3] Li W., Xu Z., Huang J.Z., Lin X.D., Luo R.C., Chen C.H., Shi P. (2014). NeuroArray: A universal interface for patterning and interrogating neural circuitry with single cell resolution. Sci. Rep..

[4] Charvet G., Rousseau L., Billoint O., Gharbi S., Rostaing J.-P., Joucla S., Trevisiol M., Bourgerette A., Chauvet P., Moulin C. (2010). BioMEA^TM^: A versatile high-density 3d microelectrode array system using integrated electronics. Biosens. Bioelectron..

[5] Frey U., Sedivy J., Heer F., Pedron R., Ballini M., Mueller J., Bakkum D., Hafizovic S., Faraci F.D., Greve F. (2010). Switch-Matrix-Based High-Density Microelectrode Array in CMOS Technology. IEEE J. Solid-St. Circ..

[6] Pickard R.P., Wall P., Ubeid M. (1990). Recording neural activity in the honeybee brain with micromachined silicon sensors. Sens. Actuat. B Chem..

[7] Aoyagi Y., Stein R.B., Branner A., Pearson K.G., Normann R.A. (2003). Capabilities of a penetrating microelectrode array for recording single units in dorsal root ganglia of the cat. J. Neurosci. Meth..

[8] Bakkum D.J., Frey U., Radivojevic M., Russell T.L., Muller J., Fiscella M., Takahashi H., Hierlemann A. (2013). Tracking axonal action potential propagation on a high-density microelectrode array across hundreds of sites. Nat. Commun..

[9] Buzsáki G. (2004). Large-scale recording of neuronal ensembles. Nat. Neurosci..

[10] Hughes M.P., Banks D.J., Ewins D.J. (2005). Action potential velocity detection using a penetrating microprobe. Meas. Sci. Tech..

[11] Ensell G.D., Banks D.J., Ewins D.J., Balachandran W., Richards P.R. (1996). Silicon-based microelectrodes for neurophysiology fabricated using a gold metallization/nitride passivation system. Microelectromech. Syst..

[12] Ensell G.D., Banks D.J., Richards P.R., Balachandran W., Ewins D.J. (2000). Silicon-based microelectrodes for neurophysiology, micromachined from silicon-on-insulator wafers. Med. Biol. Eng. Comput..

[13] Yuan F., Wiler J., Wise K., Anderson D. Micromachined Multi-Channel Microelectrodes with Titanium Nitride Sites. Proceedings of the First Joint BMES/EMBS Conference.

[14] Hughes M.P., Bustamante K., Banks D.J., Ewins D.J. Effects of electrode size on the performance of neural recording microelectrodes. Proceedings of the 1st Annual International IEEE-EMBS Special Topic Conference on Microtechnologies in Medicine and Biology.

[15] Banks D.J., Balachandran W., Richards P.R., Ewins D.J. (2002). Instrumentation to evaluate neural signal recording properties of micromachined microelectrodes inserted in invertebrate nerve. Phys. Meas..

[16] Power M.E. (1943). The brain of *Drosophila melanogaster*. J. Morphol..

[17] Chapman R. (1982). The Insects, Structure and Function.

[18] Gwilliam G., Burrow M. (1980). Electrical characteristics of the membrane of an identified insect motor neurone. J. Exp. Biol..

[19] Pearson K.R., Stein R.B., Malhotra S.K. (1970). Properties of action potentials from insect motor nerve fibres. J. Exp. Biol..

[20] Hoyle G. (1953). Potassium Ions and Insect Nerve Muscle. J. Exp. Biol..

[21] Selvakumaran J., Keddie J., Ewins D.J., Hughes M.P. (2008). Protein adsorption on materials for recording sites on implantable microelectrodes. J. Mat. Sci. Mat. Med..

[22] Bédard C., Destexhe A. (2011). Generalized theory for current-source-density analysis in brain tissue. Phys. Rev. E.

[23] Nelson J., Bosch C., Venance L., Pouget P. (2013). Microscale inhomogeneity of brain tissue distorts electrical signal propagation. J. Neurosci..

[24] Shein-Idelson M., Pammer L., Hemberger M., Laurent G. (2017). Large-scale mapping of cortical synaptic projections with extracellular electrode arrays. Nat. Meth..

[25] Wang W., Chan S.C., Heldman D.A., Moran D.W. (2007). Motor cortical representation of position and velocity during reaching. J. Neurophysiol..

[26] Paninski L., Fellows M.R., Hatsopoulos N.G., Donoghue J.P. (2004). Spatiotemporal tuning of motor cortical neurons for hand position and velocity. J. Neurophysiol..

[27] Charakopoulos A.K., Katsouli G.A., Karakasidis T.E. (2018). Dynamics and causalities of atmospheric and oceanic data identified by complex networks and Granger causality analysis. Phys. A.

